# Single-cell RNA sequencing of the mammalian pineal gland identifies two pinealocyte subtypes and cell type-specific daily patterns of gene expression

**DOI:** 10.1371/journal.pone.0205883

**Published:** 2018-10-22

**Authors:** Joseph C. Mays, Michael C. Kelly, Steven L. Coon, Lynne Holtzclaw, Martin F. Rath, Matthew W. Kelley, David C. Klein

**Affiliations:** 1 Section on Developmental Neuroscience, Laboratory of Cochlear Development, Division of Intramural Research, National Institute on Deafness and Other Communication Disorders, National Institutes of Health, Bethesda, Maryland, United States of America; 2 Molecular Genomics Core Facility, Office of the Scientific Director, Intramural Research Program, Eunice Kennedy Shriver National Institute of Child Health and Human Development, National Institutes of Health, Bethesda, Maryland, United States of America; 3 Microscopy and Imaging Core, Office of the Scientific Director, Intramural Research Program, Eunice Kennedy Shriver National Institute of Child Health and Human Development, National Institutes of Health, Bethesda, Maryland, United States of America; 4 Department of Neuroscience, Panum Institute, University of Copenhagen, Copenhagen, Denmark; 5 Office of the Scientific Director, Intramural Research Program, Eunice Kennedy Shriver National Institute of Child Health and Human Development, National Institutes of Health, Bethesda, Maryland, United States of America; University of Texas Southwestern Medical Center, UNITED STATES

## Abstract

The vertebrate pineal gland is dedicated to the production of the hormone melatonin, which increases at night to influence circadian and seasonal rhythms. This increase is associated with dramatic changes in the pineal transcriptome. Here, single-cell analysis of the rat pineal transcriptome was approached by sequencing mRNA from ~17,000 individual pineal cells, with the goals of profiling the cells that comprise the pineal gland and examining the proposal that there are two distinct populations of pinealocytes differentiated by the expression of *Asmt*, which encodes the enzyme that converts N-acetylserotonin to melatonin. In addition, this analysis provides evidence of cell-specific time-of-day dependent changes in gene expression. Nine transcriptomically distinct cell types were identified: ~90% were classified as melatonin-producing α- and β-pinealocytes (1:19 ratio). Non-pinealocytes included three astrocyte subtypes, two microglia subtypes, vascular and leptomeningeal cells, and endothelial cells. α-Pinealocytes were distinguished from β-pinealocytes by ~3-fold higher levels of *Asmt* transcripts. In addition, α-pinealocytes have transcriptomic differences that likely enhance melatonin formation by increasing the availability of the Asmt cofactor S-adenosylmethionine, resulting from increased production of a precursor of S-adenosylmethionine, ATP. These transcriptomic differences include ~2-fold higher levels of the ATP-generating oxidative phosphorylation transcriptome and ~8-fold lower levels of the ribosome transcriptome, which is expected to reduce the consumption of ATP by protein synthesis. These findings suggest that α-pinealocytes have a specialized role in the pineal gland: efficiently O-methylating the N-acetylserotonin produced and released by β-pinealocytes, thereby improving the overall efficiency of melatonin synthesis. We have also identified transcriptomic changes that occur between night and day in seven cell types, the majority of which occur in β-pinealocytes and to a lesser degree in α-pinealocytes; many of these changes were mimicked by adrenergic stimulation with isoproterenol. The cellular heterogeneity of the pineal gland as revealed by this study provides a new framework for understanding pineal cell biology at single-cell resolution.

## Introduction

The pineal gland is an essential element of vertebrate circadian and seasonal biology, acting as the source of circulating melatonin, the hormonal signal of nighttime [1]. Melatonin is synthesized from tryptophan by a four-enzyme pathway that is a highly enriched in the pineal gland. Synthesis increases at night and is associated with significant changes in many aspects of cell biology. The full extent of these rhythmic changes has become increasingly evident from the results of RNA profiling, which highlight 24-hour differences in thousands of transcripts [2, 3]. In mammals, these changes are regulated via the release of the adrenergic ligand norepinephrine from sympathetic nerve fibers pervading the gland. The daily pattern of norepinephrine release is controlled by clock cells in the suprachiasmatic nucleus (SCN), the site of the master mammalian oscillator. SCN signals are transmitted to the pineal gland via a multisynaptic pathway that passes through central and peripheral structures. Light acts on melatonin synthesis through the eyes and a retinohypothalamic projection that terminates in the SCN. Light resets the clock and gates output to the pineal gland so as to optimally entrain melatonin synthesis to the photic environment [4, 5].

While bulk RNA sequencing has profiled 24-hour rhythmic changes in the pineal transcriptome and identified pineal marker genes [2], the specific cell types exhibiting rhythmic gene expression and cell type-specific localization of these marker genes has not been established. Here, we have performed single-cell RNA sequencing (scRNA-seq) of the rat pineal gland. The goals of this study were to transcriptomically profile the cell types that comprise the gland [6–9], examine the proposal [8] that two subtypes of pinealocyte exist that differ in expression levels of *Asmt*, which encodes the enzyme that converts N-acetylserotonin to melatonin, and to determine which cell types exhibit differential gene expression between day and night. In addition, scRNA-seq can address the localization of poorly understood genes, including *Esm1* [3, 10], *Penk* [11], lipoxygenases [12, 13], and other genes required for a broad range of processing including secretion, signal transduction, and transcription. The findings presented here provide a rich foundation for a more refined level of analysis of pineal cell biology.

## Results

### Genetic profiling identifies nine cell types in the pineal gland

**Fig 1:**
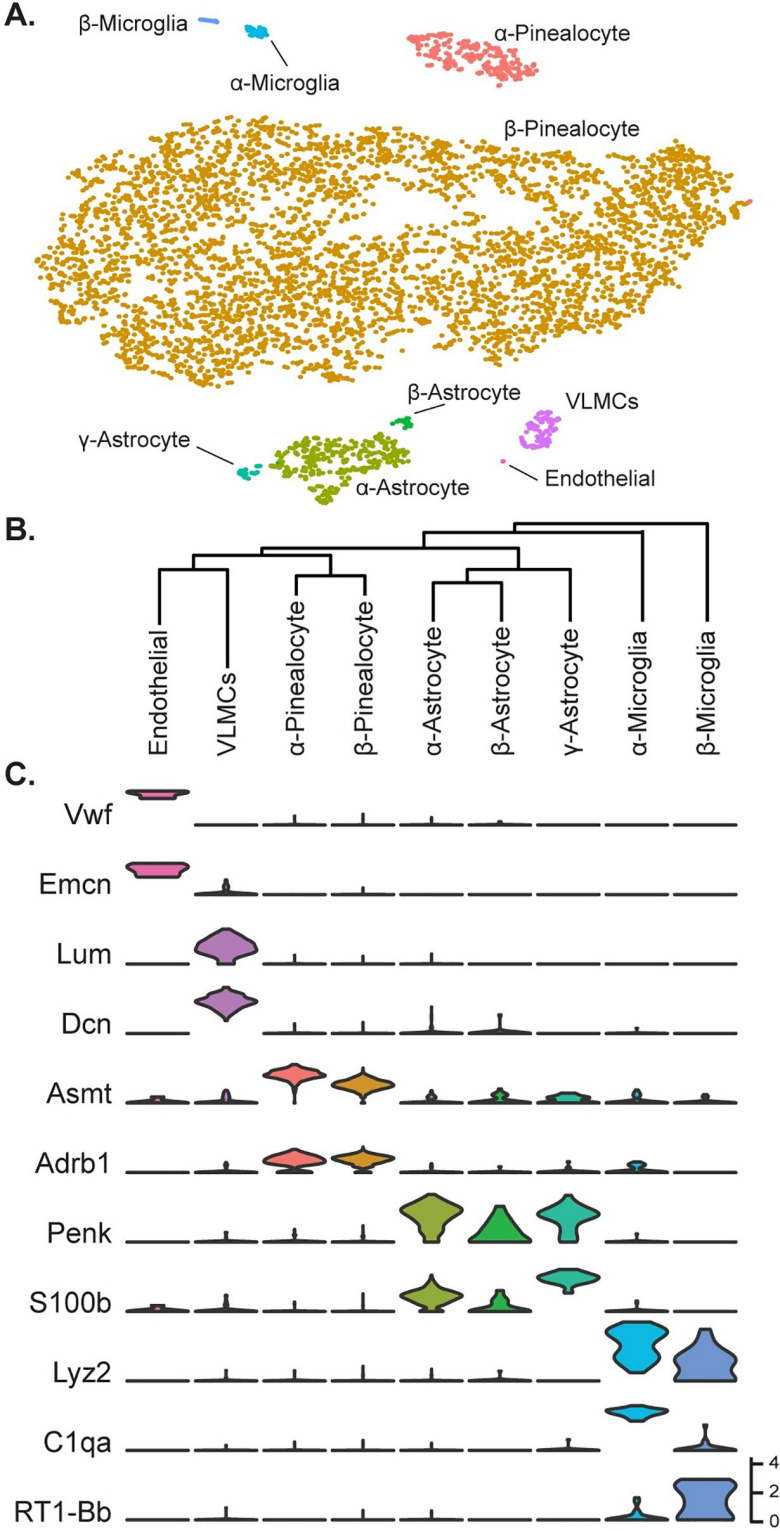
Transcriptomic characterization of cell types in the daytime rat pineal gland. (A) t-Distributed stochastic neighbor embedding (t-SNE) visualization of 5,667 daytime rat pineal gland cells profiled by scRNA-seq. Cell types are color-coded by cluster assigned from the shared nearest neighbor (SNN) clustering algorithm. (B) Hierarchical clustering dendrogram showing transcriptomic similarity of cell types, including relationships of the two pinealocyte subtypes, the three astrocyte subtypes, the two microglia subtypes, and two vascular-associated cell types: VLMCs and endothelial cells. (C) Violin plots of select marker gene expression distribution for cells from each cell type. Y-Axis is natural log of normalized counts.

To characterize the cellular heterogeneity within the pineal gland, we generated transcriptomic profiles for 5,667 single pineal gland cells from rats sacrificed six hours after lights on (Zeitgeber time (ZT0600), to mimic daytime (see Methods). Clustering analysis indicated the presence of five major cell types: melatonin-producing pinealocytes, astrocytes, microglia, vascular and leptomeningeal cells (VLMCs), and endothelial cells (Fig 1). These general designations were based on expression of established markers (Fig 1C)[14–18]. The five major cell types could be further resolved into a total of nine cell types: two populations of pinealocytes (designated as α and β), three populations of astrocytes (designated as α, β, and γ), and two populations of microglia (designated as α and β). Hierarchical clustering of the nine cell types indicates that their transcriptomic relationships are consistent with our subtype designations (Fig 1B).

**Fig 2:**
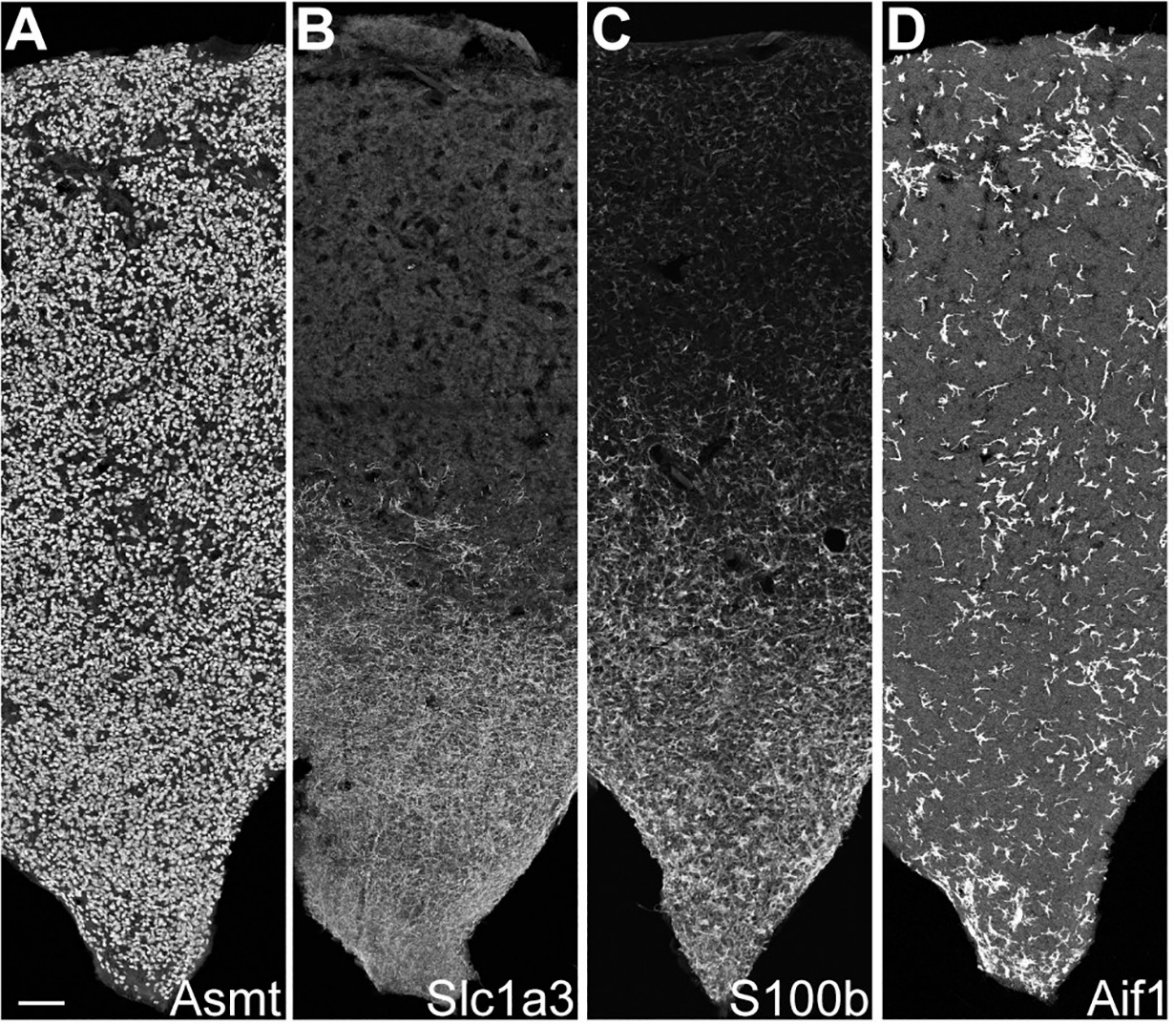
IHC reveals cell type-specific patterns of expression. Maximum intensity projections taken from IHC sections through the rat pineal gland midline with rostral stalk origin at the bottom. Images include the whole length and middle third of the width of the gland. Scale bar = 100 μm. (A) Asmt-positive pinealocytes are uniformly distributed. (B) Slc1a3-positive γ-astrocytes are most abundant in rostral region near the stalk. (C) S100b-positive cells are most abundant in the rostral region and appear elsewhere with distinctly lower density and expression strength. (D) Aif1-positive cells are unevenly distributed throughout pineal gland at low density. See S6 Fig for full images.

Pinealocytes accounted for 90% of the profiled cells (S1 Table), consistent with morphological studies [6, 7]. Genetic markers specific to pinealocytes were identified both by unsupervised analyses, i.e. Receiver operating characteristic (ROC) curve, and querying of previously identified pineal marker genes and genes in related functional groups. Such markers included *Tph1* and *Asmt*, the first and last enzymes in melatonin synthesis, respectively, and *Sag* (Fig 1C, S1 Fig)[19]. Detection of Asmt*-*positive cells by immunohistochemistry (IHC) indicated that pinealocytes were uniformly dispersed throughout the pineal gland (Fig 2A, Panel A of S6 Fig). Other genes found to be highly expressed in both pinealocyte subtypes include: *Gngt1*, *Gngt2*, *Rom1*, *Crx*, *Cngb1*, *Cnga1*, *Pde6c*, and *Slc6a6;* catecholamine receptors *Adrb1*, *Adra1b*, and *Drd4;* cholinergic receptors *Chrna3* and *Chrnb4* (Fig 1C, S1 and S2 Figs); and, a set of 49 genes expressed selectively in the pineal gland and retina [3] represented by *Sag* (S1 Fig); *Gngt1* and *Gngt2* (S4 Fig); *Crx* and *Neurod1* (S19 Fig); *Pde6b* (S15 Fig); *Drd4* (S2 Fig); and, *Cacna1f*, *Cnga1*, and *Cngb1* (S13 Fig). The localization of these transcripts has not been previously demonstrated in most cases, although they were thought to be expressed in pinealocytes based on several lines of evidence [2, 20–29].

The most highly expressed pinealocyte markers (*Tph1*, *Asmt*, *Gngt1*, and *Gngt2*) were also detected uniformly at low levels in non-pinealocytes (S1 Fig). This is likely due to contamination by ambient mRNA from lysed pinealocytes. We expect pinealocyte-derived ambient mRNA to introduce a relatively uniform and weak pinealocyte signature in nonpinealocytes because of the high proportion of pinealocytes in the preparation.

**Fig 3:**
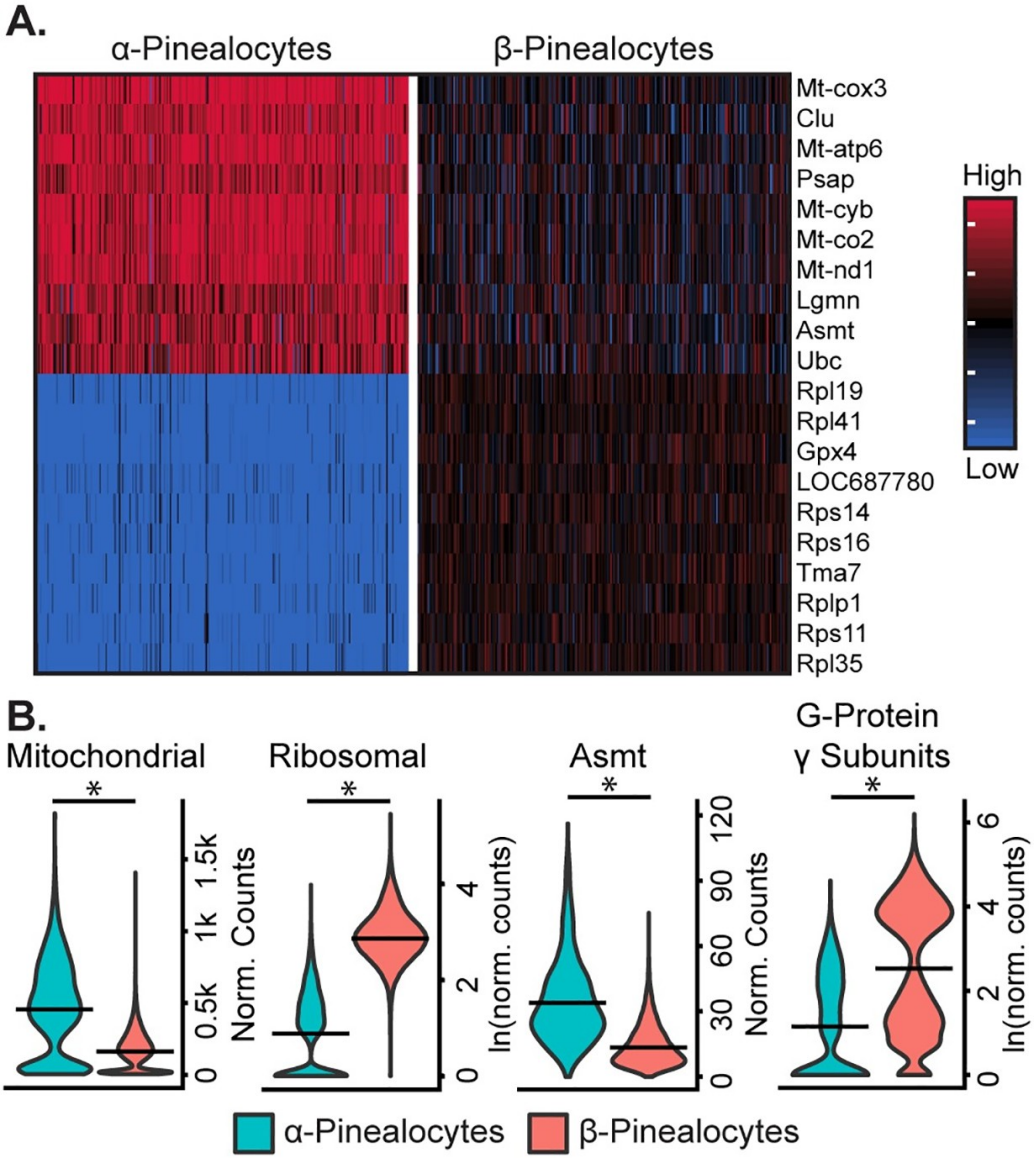
scRNA-seq reveals two transcriptionally distinct pinealocyte populations. (A) Heatmap of expression values for top 10 most differential expressed genes (by effect size) for α- and β-pinealocytes. Expression values are Z-scores of counts calculated between all cells of the two cell types. Each column represents one cell; random samples of 250 cells per cell type are shown. (B) Violin plots showing expression distribution differences between two pinealocyte subtypes for three functional groups and one gene, *Asmt*. Y-Axis is either normalized counts or natural log (ln) of normalized counts. Horizontal lines represent the mean. (*) indicates p<0.001, Wilcoxon rank sum test. All cells from each subtype are included (α-pinealocyte, n = 275; β-pinealocyte, n = 4,822). Mitochondrial group includes differentially expressed mitochondrial OxPhos genes (p<0.05, fold change ≥2.0), ribosomal group includes top 20 most differential ribosomal genes by effect size (p<0.05, fold change ≥2.0), G-protein γ-subunits include *Gngt1*, *Gngt2*, *Gng10*, and *Gng13* (see S5 Fig for individual genes).

α- and β-Pinealocytes accounted for 5% and 95% of pinealocytes, respectively. Whereas these two cell types express an overlapping set of marker genes, comparison of their transcriptomes by differential expression analysis indicated distinct differences in expression of specific genes and sets of functional groups. The most prominent differentiating genes as ranked by effect size included *Asmt*, genes involved in mitochondrial oxidative phosphorylation (OxPhos), ribosomal genes, and G-protein γ-subunits (Fig 3, S1, S3 and S4 Figs). α-Pinealocytes had 3.4-fold greater average expression of *Asmt* (Fig 3B), consistent with previous IHC evidence of marked cell-to-cell differences in Asmt protein[8]. Transcript counts from subsets of the mitochondrial OxPhos and ribosomal protein transcriptomes were respectively pooled for analysis (S5 Fig). α-Pinealocytes had a 2.3-fold greater average expression of the eight differentially expressed OxPhos genes, and 8.2-fold lower average expression of the top 20 ranked differentially expressed ribosomal genes. Additionally, α-pinealocytes had 5.4-fold lower average expression of G-protein γ-subunits *Gngt1*, *Gngt2*, *Gngt10*, and *Gng13* than α-pinealocytes (Fig 3B, S4 Fig).

**Fig 4:**
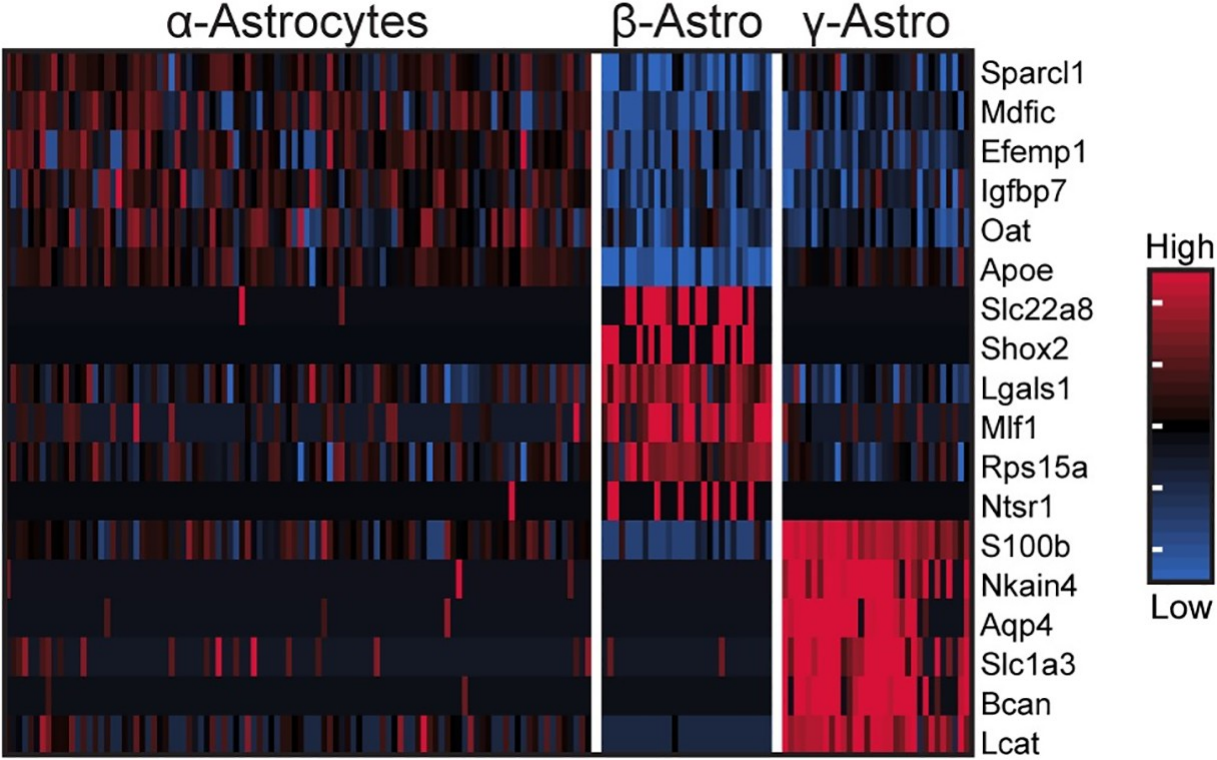
scRNA-seq reveals three transcriptionally distinct astrocyte populations. Heatmap of expression values for top 6 most differential expressed genes (by effect size) for α-, β-, and γ-astrocytes. Expression values are Z-scores of counts calculated between all cells of the three cell types. Each column represents one cell; random sample of 100 cells from α-astrocytes are shown; all β- and γ-astrocytes are shown. See also S1 Fig.

Astrocytes accounted for 7% of profiled cells (S1 Table) and were identified based on expression of glial markers including *Aldh1a1*, *S100b*, and *Tnfrsf21* (Fig 1C, S1 Fig)[14–16]. These cells also had high expression of *Penk*, *Apoe*, and *Esm1* (Fig 1C, S1 Fig). α-, β-, and γ-Astrocytes accounted for 85%, 7%, and 8% of astrocytes, respectively. Differential expression analysis revealed that α-astrocytes exhibited higher expression of *Sparcl1*, *Mdfic*, *Efemp1*, *Oat*, and *Gad2* as compared to the other astrocytes. β-Astrocytes exhibited higher expression of *Slc22a8*, *Shox2*, *Lgals1*, and *Mlf1*. γ-Astrocytes exhibited higher expression of *S100b*, *Nkain4*, *Aqp4*, *Slc1a3*, *Bcan*, and *Gfap* (Fig 4, S1 Fig). IHC detection of Slc1a3-positive cells indicated that γ-astrocytes were generally limited in distribution to the rostral region of the gland close to the pineal stalk (Fig 2B, Panel B of S6 Fig). Gfap protein was also exclusively detected in the same region(Panel C of S6 and S7 Figs), consistent with previous observations [30–32]. scRNA-seq indicated that *S100b* was expressed in all astrocyte subtypes, but most strongly in γ-astrocytes (Fig 4, S1 Fig). IHC detection of S100b-postive cells indicated that astrocytes are dispersed throughout the gland, though higher expression is detected in the rostral region, consistent with the higher expression *S100b* exhibited by γ-astrocytes (Fig 2C, Panel D of S6 and S7 Figs).

Microglia accounted for 1% of profiled cells (S1 Table) and were identified by expression of *Aif1* and *Lyz2* (Fig 1C, S8 Fig)[14–16]. IHC detection of Aif1-positive cells indicated that microglia were distributed throughout the gland (Fig 2D, Panel E of S6 Fig). α- and β-Microglia accounted for 64% and 36% of microglia, respectively. They were differentiated based on the expression of genes linked to immune function: α-Microglia were enriched with complement subcomponents *C1qa*, *C1qb*, and *C1qc*, whereas β-microglia were enriched with MHC Class II genes *RT1-Da*, *RT1-Db1*, and *RT1-Ba* (S8 Fig).

Vascular cells, including endothelial cells and vascular and leptomeningeal cells (VLMCs) comprised the remaining profiled cells. VLMCs accounted for 2% of profiled cells, identified by expression markers *Lum*, *Dcn*, *Col1a1*, and *Gjb2* (Fig 1C, S9 Fig)[33]. These cells also exhibited high expression of *Cdh11* (S9 and S10 Figs). Endothelial cells accounted for 0.1% of profiled cells and were identified by expression of *Vwf*, *Esam*, *Cdh5/VE-cadherin*, and *Ecmn* (Fig 1C, S9 and S10 Figs)[14–18].

Examination of the expression patterns of gene families and functionally related genes revealed cell-specific expression patterns for catecholamine and cholinergic receptors (S2 Fig), glutamate and GABA signaling (S11 and S21 Figs), purinergic receptors (S12 Fig), G-protein subunits (S4 Fig), cadherins and gap junction elements (S10 Fig), calcium channel subunits (S13 Fig), potassium channel subunits (S14 Fig), cyclic AMP signaling proteins (S15 Fig), Tnfa signaling proteins (S18 Fig), ephrin signaling elements (S17 Fig), circadian clock elements (S22 Fig), lipoxygenases and phospholipases (S23 Fig), aquaporins(S20 Fig), secretion-related proteins (S16 Fig) and transcription factors (S18 Fig).

### ScRNA-seq reveals day/night changes in pineal cell transcriptomes

**Fig 5:**
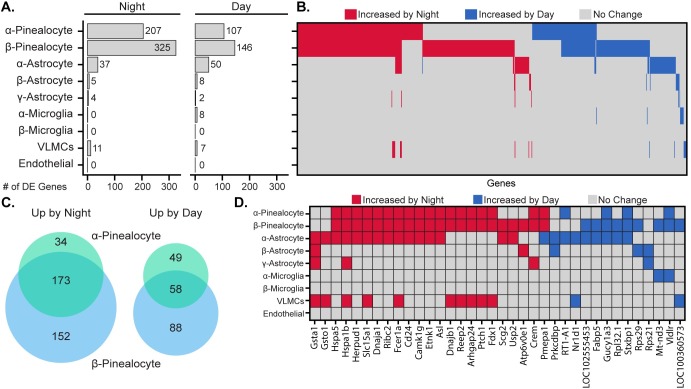
Changes in gene expression between day and night occur in a cell type-specific manner. (A) Number of differentially expressed (DE) genes upregulated by night or day by cell type. A gene is considered DE at p<0.01 (Wilcoxon rank sum), when expressed in at least 15% of cells in either of the two samples being tested, fold change ≥2.0, and effect size ≥0.35. (B) Heatmap summary of all 644 DE gene changes by cell type. Each column represents one gene. (C) Venn diagram of number of overlapping DE genes in α- and β-pinealocytes by day and night. (D) Heatmap summary of DE genes found in at least one non-pinealocyte and one other subtype. See also dot plots in SI.

To examine expression differences between day and night in specific pineal cell types, scRNA-seq profiles of 7,940 pineal gland cells from rats sacrificed at night (ZT1800) were generated and compared to profiles of the daytime cells characterized above. Clustering analysis of the night cells yielded the same cell types and proportions found in the day cell population (S1 Table, S24 Fig). The number of significantly differentially expressed genes between day and night varied considerably among cell types (Fig 5). Pinealocytes exhibited the greatest degree of differential expression, with 359 genes upregulated at night and 195 genes upregulated during the day. Differentially expressed genes included *Aanat*, *Crem*, *Drd4*, *Pde10a*, and others previously established to exhibit day/night differential expression [2]. β-Pinealocytes had 1.5-fold more differentially expressed genes overall than α-pinealocytes, although there was considerable overlap, as 173 and 58 of the same genes were upregulated in both pinealocyte populations during night and day, respectively (Fig 5C). Among non-pinealocytes, α-astrocytes had the greatest degree of differential expression, with 37 genes increasing at night and 50 increasing during the day. Other non-pinealocytes had comparatively fewer differentially expressed genes, several of which overlapped between different cell types (Fig 5D). *Aanat* is highly expressed in pinealocytes at night and was also detected uniformly at low levels in non-pinealocytes (S1 Fig), as addressed above, this is likely due to contamination by pinealocyte-derived ambient mRNA. Because this can make non-pinealocytes erroneously appear to differentially express *Aanat*, the gene was removed from analysis for non-pinealocytes. *Pmepa1* was the only gene found to be both upregulated in a cell type (α- and β-pinealocytes) at night and upregulated in another type (α-astrocytes) during the day (Fig 5D).

### Isoproterenol treatment mimics day/night changes in the transcriptomes of pinealocytes

**Fig 6:**
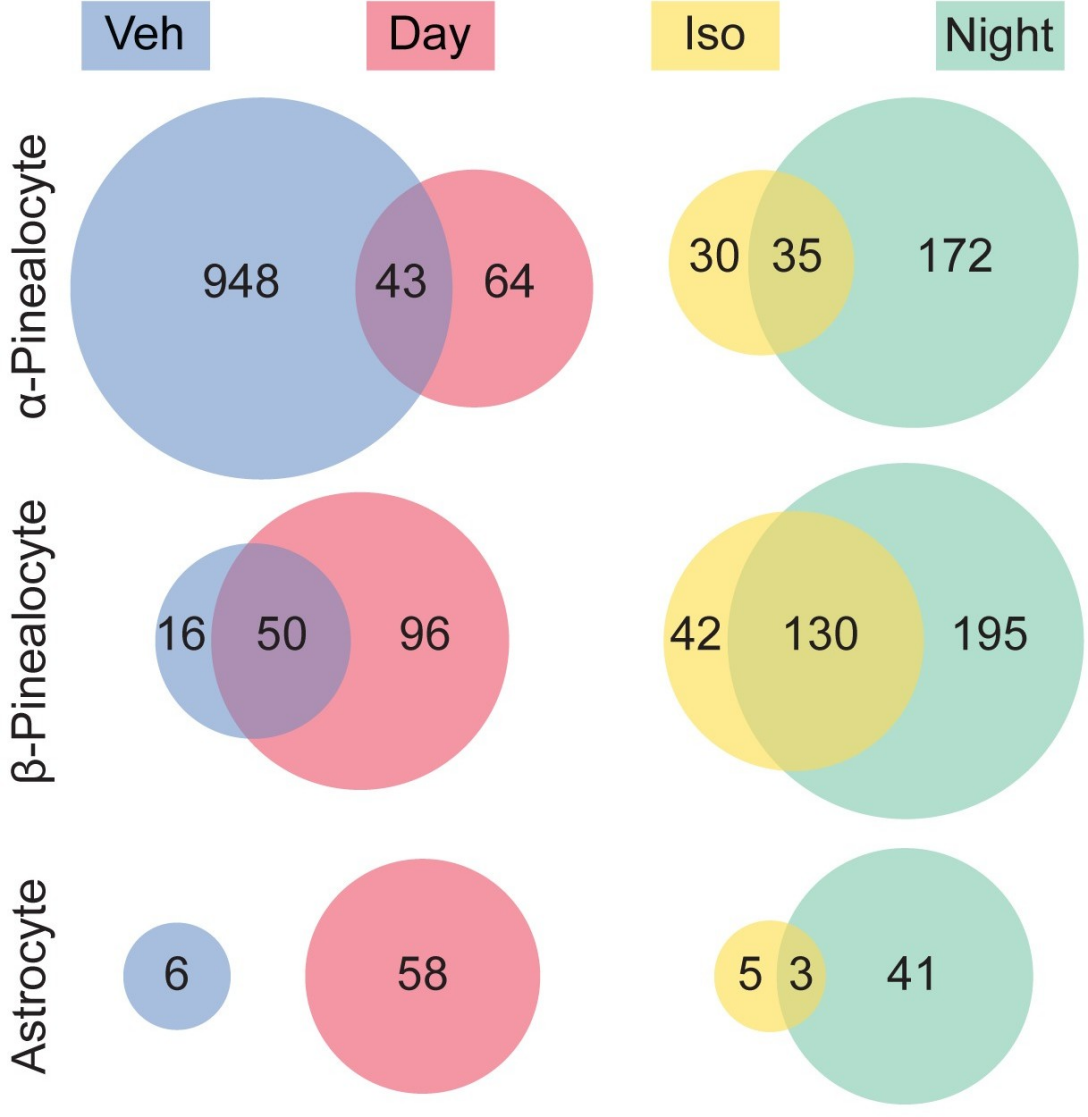
Comparison of differentially expressed genes between the nighttime pineal gland and isoproterenol-treated pineal gland. Venn diagrams indicate the number of genes that were found to be significantly differentially expressed (DE) in the pineal gland (see methods). There was overlap between DE genes upregulated at night and by isoproterenol (Iso) treatment, as well as overlap between DE genes upregulated during the day and upregulated in the vehicle control treated (i.e. downregulated by isoproterenol treatment), in 3 cell types. Other cell types are not shown.

Many changes in the pineal transcriptome seen at night are mimicked by treatment with the β-adrenergic agonist isoproterenol [2, 3]. Here we compared the effects of isoproterenol treatment to day/night changes in the transcriptome of individual pineal cell types by generating scRNA-seq profiles of 1,996 pineal cells (Fig 6). The same general cell types were identified as above but, due to a reduced number of cells, it was not possible to resolve astrocytes and microglia into subtypes. Differential expression analysis indicated that 99% of the transcriptional changes observed following isoproterenol treatment occurred in α- and β-pinealocytes, consistent with the evidence that these are enriched with β-adrenergic receptors that mediate effects of neural stimulation by norepinephrine. The remaining 1% of changes occurred in astrocytes; isoproterenol treated microglia, VLMCs, and endothelial cells had no differentially expressed genes. 54%, 76%, and 38% of genes upregulated following isoproterenol treatment were also upregulated at night in α-pinealocytes, β-pinealocytes, and astrocytes, respectively. 4% and 76% of genes were downregulated following isoproterenol treatment (i.e. upregulated following vehicle control treatment) in α-pinealocytes and β-pinealocytes, respectively.

## Discussion

Using single-cell RNA-seq analysis, we were able to characterize the transcriptomes of nine cell types in the pineal gland and identify expression differences occurring between day and night. These results expand on previous evidence which was interpreted to reflect the existence of two types of pinealocytes with different roles in melatonin synthesis [8]. In addition, transcriptomic profiling of non-pinealocytes is of special value in light of the growing evidence that astrocytes and microglia are important elements for regulating pinealocytes and pineal function [34–37]. Taken together, these results characterize the pineal gland as an integrated team of highly specialized cells dedicated to melatonin production.

### Pinealocytes

**Fig 7:**
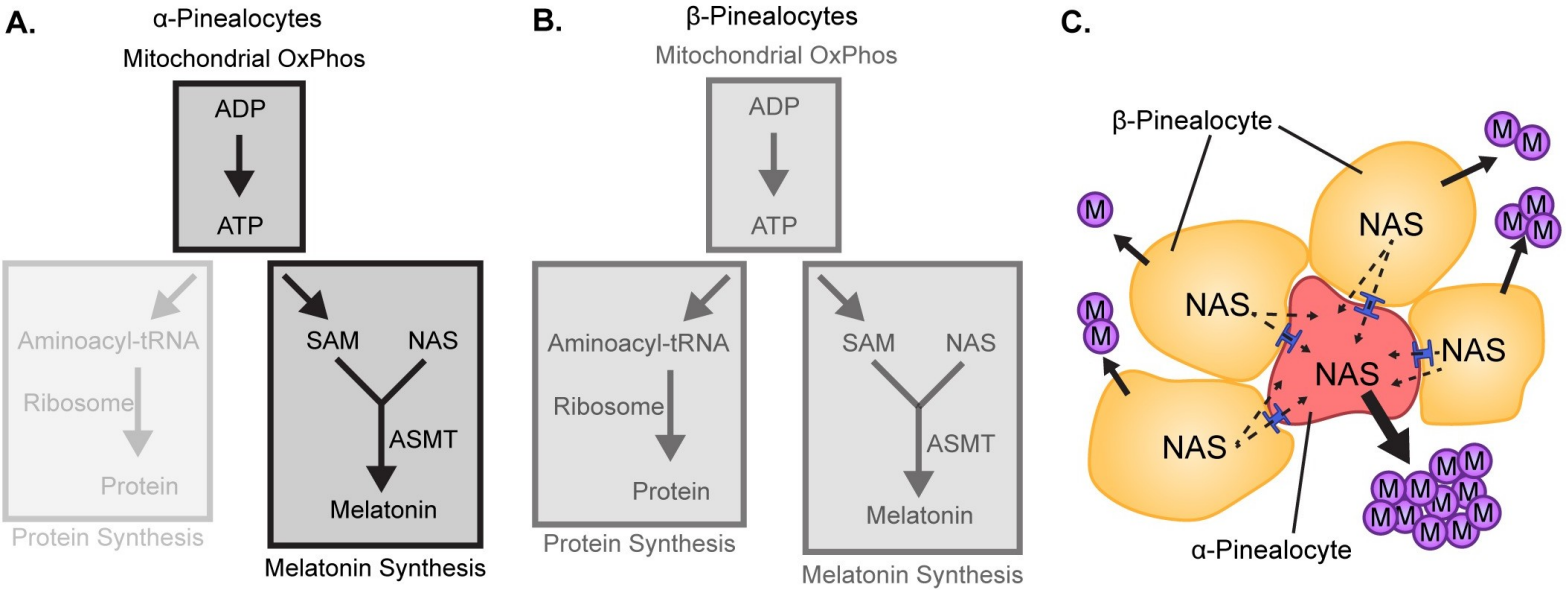
Differences in melatonin synthesis between α- and β-pinealocytes inferred from scRNA-Seq. **(**A-B) Opacity indicates relative strength of the pathway module; greater opacity indicates a more active pathway. (A) Conversion of N-acetylserotonin (NAS) to melatonin in α-pinealocytes is enhanced by increased ASMT activity and increased S-adenosyl methionine (SAM) availability, which is increased by greater ATP availability. ATP availability is increased by elevated ATP production from oxidative phosphorylation (OxPhos), as inferred by greater expression of mitochondrial genes in α-pinealocytes. ATP availability is also increased by reduced consumption by protein synthesis, as inferred by decreased expression of ribosomal genes in α-pinealocytes. (B) β-Pinealocytes also undergo melatonin synthesis, but do not have the same production increasing enhancements as α-pinealocytes. (C) Melatonin (M) is synthesized in both pinealocyte subtypes from N-acetylserotonin (NAS). NAS that is not converted to melatonin in β-pinealocytes enters the α-pinealocyte by passive diffusion through membranes and gap junctions (shown in blue). NAS is subsequently converted to melatonin by the high efficiency Asmt system in the α-pinealocyte, thereby maximizing melatonin production.

The finding of 3.4-fold greater expression of *Asmt* in α-pinealocytes supports previous claims of two pinealocyte subtypes differentiated by Asmt immunoreactivity [8]. The elevated *Asmt* expression suggests that these cells have an enhanced capacity to catalyze the formation of melatonin from N-acetylserotonin. scRNA-seq also revealed four other features that differentiate α- and β-pinealocytes. α-Pinealocytes exhibited higher expression of the OxPhos transcriptome, reduced expression of both *RP* and *Gngt* genes, and a generally suppressed nocturnal increase in gene expression. We hypothesize that these differences may act together to further enhance melatonin production at the Asmt step by increasing availability of the Asmt cofactor SAM, as detailed below (Fig 7). SAM is synthesized from ATP and methionine.

We expect that ATP availability in α-pinealocytes will be increased by the differences detailed above as follows: ATP production is likely to be elevated by the 2.3-fold greater expression of the OxPhos transcriptome. Second, ATP availability is likely to be further enhanced by the 8.2-fold lesser expression of the RP transcriptome, leading to decreased consumption of ATP by protein synthesis. Third, the 5.4-fold lesser expression of *Gngt* genes will suppress nighttime increases in G-protein-dependent enhanced gene expression resulting in reduced ATP utilization for time-of-day changes in protein synthesis (Fig 7). These features, along with greater *Asmt* expression, appear to establish a unique role for the α-pinealocyte as a specialized cell that enhances the overall efficiency of the pineal gland to convert tryptophan to melatonin by increasing the likelihood that N-acetylserotonin is converted to melatonin. According to this interpretation, these cells could act on N-acetylserotonin synthesized in either α-pinealocytes or β-pinealocytes. The transfer of N-acetylserotonin from β-pinealocytes to α-pinealocytes is highly likely reflecting the highly lipophilic nature of N-acetylserotonin, in addition to the presence of gap junctions [38] and expression of common cadherins in α- and β-pinealocytes (Fig 7, S10 Fig).

Based on this, one can reasonably predict that the relative abundance of the two subtypes will influence the amount of melatonin produced by the pineal gland. Differences in the relative abundance of these cells could contribute to differences in circulating melatonin levels among individuals and as a function of age and other factors.

A concern with the concept of two functionally different pinealocyte subtypes is whether the biology of α-pinealocytes is compromised by insufficient resource allocation and reduced protein synthesis. However loss of some functions, such as maintenance of extracellular matrix, would be compensated for by β-pinealocytes. Similarly, essential factors not synthesized in the α-pinealocytes could be provided by the β-pinealocyte through transfer via gap junctions, membrane permeability, and import/export mechanisms. These proteins may be sufficiently stable that a lack of production on the part of α-pinealocytes might not have adverse effects on these cells. Moreover, decreased activity of some pathways in the α-pinealocytes may further enhance Asmt activity by eliminating potential inhibitors and promoting a more favorable cellular environment for Asmt catalysis. Accordingly, the two pinealocyte subtypes might function in a way in which resources and metabolic pathways are shared.

The existence of two pinealocyte subtypes leads to the question of the regulation of each population. One possible explanation is that distinct phenotypes are established early in development and represent end-stage differentiated cells; their relative abundance might reflect selective cell death and replacement. Alternatively, it is possible that the α- and β-pinealocytes can change phenotype during life, perhaps in response to hormonal or neural signals. As such, this might represent a reversible mechanism to fine tune melatonin production.

Whereas the transcriptomic evidence of two pinealocytes is convincing, it is not clear how this body of evidence relates to morphological observations of the existence of two pinealocytes, identified as light and dark pinealocytes and alternatively as Type 1 and Type 2 pinealocytes [6, 9]. As β-pinealocytes account for 95% of pinealocytes, Type 1 pinealocytes account for 90% to 95% of pinealocytes. Morphological studies identified differences in Type 1 and 2 pinealocyte features associated with secretion. However, the transcriptomic evidence of these features does not strongly differentiate α- from β-pinealocytes, as both pinealocyte subtypes express similar levels of members of the secretion-associated granin family (*Chga*, *Scg2*, *Scg5*, *Scg3*, *and Chgb*) (S16 Fig). Moreover, it is also possible that the morphologically distinct pinealocytes are β-pinealocyte subtypes. Efforts to resolve this question are likely to benefit from the use of scRNA-seq data to select targets for morphological studies designed to differentiate the two types of pinealocytes.

A puzzling feature of the pineal gland relates to expression of lipoxygenase *Alox15*, which is expressed at higher levels in the pineal gland relative to other tissues [2, 3]. The current studies extend this by determining that *Alox15* is primarily expressed in both pinealocytes (S23 Fig). *Alox15* is of interest because it is involved in the synthesis of hepoxilins, which are produced in abundance by this tissue [39–41]. Pineal hepoxilins have been generally ignored in the last two decades. They influence inflammation, blood flow, and pain perception [42, 43] and could act within the pineal gland or at extrapineal sites. Hepoxilins are synthesized by both Alox12 and Alox15[44, 45]. The expression of *Alox15* in the pineal gland, but not *Alox12* (S23 Fig) [2, 3], indicates that Alox15 is responsible for hepoxilin formation in this tissue.

### Non-pinealocytes

In contrast to the unique nature and limited distribution of pinealocytes, non-pinealocytes represent widely-distributed cell types. Astrocytes and microglia particularly exhibit distinct molecular and morphological heterogeneity, reflecting a range of phenotypes. Here, we identified three transcriptionally distinct astrocyte subtypes (Figs 1 and 4, S1 Fig). All subtypes shared expression of *Esm1*, *Apoe*, and *S100b*, albeit at varying levels. This variation is consistent with previous studies on pineal astrocytes, which have reported immunocytochemical differences in staining of astrocyte marker proteins [30, 31, 46]. The evidence of differential anatomical distribution, particularly of γ-astrocytes located at the most rostral portion of the pineal gland near the pineal stalk, along with the distinct transcriptomic differences, suggests unrecognized functional differences among the astrocyte subtypes. This is supported by evidence of different day/night expression patterns among astrocytes (Fig 5), with α-astrocytes having the most differentially expressed transcripts.

The functional roles of GABA and glutamate in the pineal gland remain an area of active study, as regards the roles they play in controlling melatonin synthesis [34, 47–50]. Our studies document expression in pinealocytes and astrocytes and expand on current knowledge of the distribution of genes that mediate GABA and glutamate signaling by detailing the subtypes in which they are expressed (S11 Fig), introducing a new layer of complexity.

Previous work has established *Esm1* is expressed in the pineal gland in nonpinealocytes [3, 10]. The identification of astrocytes as *Esm1*-expressing cells in the pineal gland, and not endothelial cells [51], raises the issue of functionality. Astrocyte-derived *Esm1* may play a role in vascularization of the pineal gland, based on studies in other systems [52].

Pineal microglia have been well-studied [7, 53] and have recently been found to play a role in pruning during pineal development [35]. Pruning of synapses in the brain during development is thought to involve complement-tagging of targets for phagocytic removal [54, 55]. Our finding that α-microglia expresses complement subcomponents (S8 Fig) provides evidence of a local source of complement to mark material for removal [56]. Removal of the opsonized material may involve the β-microglia subtype, characterized by expression of MHC-Class II members *RT1-Da*, *RT1-Db1*, and *RT1-B*a (S8 Fig). The differing roles of these cell types are highlighted by other genetic differences, including exclusive expression of *Chrne* in β-microglia (S2 Fig), suggesting that cholinergic input might play a role in controlling these cells.

The identification of VLMCs [33] in this study introduces these cells to pineal gland literature. VLMCs exhibit day/night differential gene expression (S9 Fig), which may reflect nocturnal adrenergic stimulation mediated by α_2a_-adrenergic receptors, encoded by *Adra2a*, the only adrenergic receptor gene strongly expressed in this cell type (S2 Fig). Day/night transcriptomic differences in these cells are likely to reflect the effects of release of norepinephrine, a mixed α/β-adrenergic agonist. The failure of isoproterenol to mimic day/night transcriptomic changes in VLMCs may reflect the strong β-adrenergic nature of the drug and the highly α-adrenergic selectivity of the α_2a_-adrenergic receptor.

Endothelial cells were found to selectively express *Adcy4*, pointing to the involvement of cyclic AMP in their biology (S15 Fig). Another finding of interest is scRNA-seq evidence for a paracrine endothelial-to-astrocyte Tnfa-based signaling system in which the Tnfa ligand *Tnfsf10* is released from endothelial cells and binds to the Tnfa receptor *Tnfrsf21* on astrocytes. Similarly, an endothelial-to-pinealocyte paracrine relationship may exist, suggested by the expression of the ephrin ligand *Efna1* in the endothelial cells and receptors in the pinealocytes.

A feature of these data is the apparent contamination of the non-pinealocytes cells by ambient mRNA from lysed pinealocytes. It is possible that this issue could be minimized or eliminated by using mRNA sequencing of single nuclei instead of single cells in order to reduce contaminants from pinealocyte cytoplasm. However, it is possible that this approach would eliminate detection of weakly expressed transcripts whose detection is dependent upon their accumulation in the cytoplasm. This approach will be considered in future studies.

### Day/night differences in gene expression

Whereas 24-hour changes in pineal gland gene expression are controlled by an autonomous molecular circadian clock in all vertebrates, the location of the clock is different among vertebrate classes. The molecular circadian clock driving the mammalian pineal gland is in the SCN, as indicated above. In contrast, the molecular clock that drives pineal rhythms in birds and fish is in pineal cells. Here, scRNA-seq revealed cell type-specific differential expression of some clock genes between day and night, including Cry2 in both pinealocyte subtypes, Rorb in β-pinealocytes and Dbp, Per3, Tef, Arntl, and Nr1d1 in α-astrocytes (S24 Fig). This raises interesting questions regarding the roles these daily expression changes play in pineal biology and whether they are regulated by the SCN. As well as whether these changes reflect the influence of the autonomous circadian clock or if they act as a local mechanism that influences the day/night changes driven by the SCN.

### Final statement

scRNA-seq has revealed the complex cellular composition of the pineal gland. The evidence of two transcriptomally distinct pinealocytes offers a new perspective on the understanding of the melatonin synthesis process. scRNA-seq has also identified the cell-specific nature of day/night differences in gene expression previously characterized by bulk RNA sequencing [2]; although the majority of the changes occur in pinealocytes and are controlled by an adrenergic mechanism, it is clear that other cells exhibit day/night changes in gene expression. This report provides a new outline of pineal gland composition and function and points to previously unrecognized cellular functions, expression patterns, and cell:cell paracrine control mechanisms. These advances enrich our understanding of pineal cell biology reflecting the interactions of the transcriptionally distinct pinealocytes, astrocytes, microglia, and vascular cells, each with different roles organized to optimize melatonin production.

## Materials and methods

### Biological materials

Male and female Sprague Dawley rats (Taconic Farms, Germantown, NY) used for all studies were maintained in facilities at the National Institutes of Health. Animal use and care was in accordance with National Institutes of Health guidelines. The lighting cycle was 12 hours light, 12 hours dark. They were housed in rooms that provided approximately 325 lux at cage level; room temperature was approximate 23° C. Animals were bred in house, were 250 to 300 grams at time of usage and had been housed in groups of three for two weeks prior to usage. The temperature and room illumination were the same for all animals. Euthanization at night was done with light generated by a head mounted or hand held flash light covered with 600 nm cut-off red filters (Apollo Design Technologies, Fort Wayne, IN). The exposure to this light was minimal.

Tissue for single-cell RNA sequencing was obtained in two experiments. Pools of glands were composed of tissue obtained from two males and two females. For pineal gland characterization and day/night differential expression analysis, two groups of animals were CO_2_-anesthetized at 6 hours after lights on (Zeitgeber (ZT)0600) (day) and two groups at 6 hours after lights off (ZT1800) (night); pineal glands were rapidly removed and placed into DMEM culture medium (4°C). For adrenergic stimulation and differential expression analysis, one group of animals received a subcutaneous injection of 10 mg/kg isoproterenol (Iso) (Sigma-Aldrich; St. Louis, MO) dissolved in phosphate buffered saline (PBS) at ZT0200 and ZT0400; the control group received vehicle alone. Animals were CO_2_-anesthetized at ZT0600 and tissue was obtained as described above.

To obtain pineal glands for immunohistochemical analysis, rats were anesthesized with isofluorane and perfused (gravity feed, 3 to 4 min) via the left ventricle with phosphate buffered saline (PBS), pH 7.4 containing 137 mM NaCl, 10 mM phosphate (KH_2_PO_4_ + Na_2_HPO_4_) to clear blood and then with 100 mL of 4% paraformaldehyde in PBS to fix the tissue. Perfusion was conducted between ZT0200 and ZT0700. Pineal glands and brains were post-fixed for 24 hours in the same fixative prior to dissection. All tissues were cryoprotected in 30% sucrose 24 to 48 hours at 4°C, then embedded in Tissue Tek O.C.T. compound (EMS; Hatfield, PA) and stored at -80°C.

Single cells were obtained using the Papain Dissociation System (Worthington; Lakewood, NJ) according to the manufacturer’s instructions, modified for pineal cells [57] as follows: A 5 mL volume of papain solution was prepared in the supplied EBSS and equilibrated in an incubator with 5% CO_2_ at 37°C for at least 30 minutes. Immediately prior to the initiation of papain digestion, 1 mL of solution was added to the DNase vial, mixed gently, and recombined. To initiate dissociation, 2.5 mL of papain/DNase solution was added to each set of 4 glands in a 35-mm petri dish and incubated at 37°C with intermittent agitation. After 50 minutes, the glands were gently triturated using a 1 mL pipette tip and incubated for an additional 10 minutes. The glands were triturated again at 5-minute intervals until dissociation was judged to be nearly complete by visual examination. When dissociation was complete, cells were transferred to a 15 mL tube; the dissociation petri dish was rinsed with 2.5 mL of DMEM, which was added to the 15 mL tube. A 2.5 mL volume of the supplied protease inhibitor solution was added, the cells were collected by centrifugation (100 x g, 5 min, 4°C) and then gently resuspended in 0.5 mL of DMEM. 0.5 mL of protease inhibitor was added to the cell suspension. Clumps and debris were removed by passing the suspension through a pre-wetted 20 μm strainer (PluriSelect; San Diego, CA); the strainer was rinsed with 5 mL DMEM. A 1.5 ml aliquot of cells was then transferred to a microfuge tube and centrifuged (100 x g, 5 min, 4°C). Cells were resuspended in 1 mL of PBS containing 0.04% bovine serum albumin and counted. The final cell concentration was 300 to 500 single cells per μL.

### Single-cell mRNA sequencing

Cells were captured using a Chromium Controller (10X Genomics; Pleasanton, CA) and single-cell cDNA libraries were prepared using Chromium Single Cell 3′ Reagent Kits v2 following the manufacturer’s instructions. Libraries were sequenced on an Illumina HiSeq2500 (Illumina; San Diego, CA), generating 98 bp of sequence adjacent to the polyA tail. In one experiment, two biological replicates each of day pineal cells and night pineal cells were analyzed (S1 Table). Groups of 2,400 to 4,300 cells per sample were recovered, with 40 to 70k reads per cell and 2,700 to 3,000 genes per cell detected on average. In another experiment, single groups of pineal cells from isoproterenol-treated animals and from vehicle treated animals were analyzed. Groups of 1,500 to 2,000 cells per sample were recovered, with 63 to 71k reads per cell and 2,600 to 2,900 genes per cell detected on average.

Sequenced single-cell libraries were analyzed by generating gene-level counts using the CellRanger analysis software v2.1.0 (10X Genomics) to align sequencing reads to the rat Rnor6.0 reference genome (Ensembl). Low-quality cells were automatically removed during initial alignment if they expressed fewer than 800 genes; genes detected in fewer than 3 cells were excluded from the analysis. Cells with outlier numbers of unique molecular identifiers (UMIs) (>25–35,000) were also removed to exclude possible cellular doublets; cutoffs were specific to each sample. The two biological replicates under day conditions were pooled and subsequently analyzed as one sample (n = 5,667); the two biological replicates for night were similarly pooled (n = 7,940). Visualization of the pooled day samples and pooled night samples did not indicate appreciable batch effects (S24 Fig). Dimensional reduction analysis was performed using the Seurat v2.2.0 package for R [58]. Gene counts were normalized to 10,000 molecules per cell. FindVariableGenes (parameters: x.low.cutoff = 0.0125, x.high.cutoff = 3, y.cutoff = 0.3) was used to identify lists of ~1,500 highly variable genes for the day and night samples. RunPCA was run using the variable genes lists to compute principal components (PC). PC analysis results were projected onto the remaining genes using ProjectPCA.

Cells were clustered using a shared nearest neighbor (SNN)-based algorithm through FindClusters, using the top 13 (day), 10 (night), 8 (vehicle control), and 7 (isoproterenol) PCs. The clustering results were visualized by t-distributed stochastic neightbor embedding (t-SNE) through RunTSNE (parameters: do.fast = TRUE) which generates 2D projections of the cells with clusters color-coded according to the output from FindClusters. Cluster identities were determined using established marker genes. In each sample, the population of β-pinealocytes was embedded on the t-SNE plot as one large cluster, but was split into smaller color-coded clusters by the SNN clustering algorithm. These color-coded clusters were consolidated into one large cluster for subsequent analyses to match the t-SNE embedding. The population of endothelial cells in the day sample did not cluster independently from the VLMCs but were embedded separately from the VLMCs in the t-SNE plot; their identity was set manually. Putative cellular doublets were removed by excluding cells expressing moderate-to-high levels of genes that were particularly specific to separate clusters (day, n = 60; night, n = 125).

Initial analysis of the sequencing reads indicated that there were little-to-no detected transcripts for some genes known to be pineal-enriched based on bulk RNA-sequencing [2] (https://snengs.nichd.nih.gov/) and that reads for these genes were aligning downstream of their respective annotated 3’ UTRs. Accordingly, annotations for these genes (S3 Table) were extended to cover these reads, as described [59]. Gene count values were determined based on the extended genome for cells that remained part of the data set after QC and clustering. The counts were re-normalized; the cells were not re-clustered. The adjusted values were used for differential expression analysis and visualization (excluding t-SNE plots). Cell type-specific markers were predicted using the FindMarkers Seurat function, set to ‘wilcox’ or ‘roc’, and evaluated manually using Seurat’s visualization functions. Differential expression analysis was performed as follows: Each of the nine cell populations identified were tested for differential expression between the night and day conditions using the Wilcoxon Rank Sum test, run through the FindMarkers Seurat function with filtering parameters set to 0. All 15,744 detected genes were tested. For the isoproterenol experiment, each of the five cell populations were tested for differential expression between the isoproterenol and vehicle treated conditions as above; all 13,984 detected genes were tested. P-values were adjusted using the False Discovery Rate adjustment at α = 0.05. Genes were considered differentially expressed at p<0.01, when expressed in at least 15% of cells in either of the two samples being tested, fold change ≥ 2.0, and effect size ≥ 0.35, to prevent false positives from technical noise in low expressing genes [60].

Fold change was calculated by

$$\frac{{0.01+\bar{x}}_{night}}{{0.01+\bar{x}}_{day}}$$

or the inverse, and similarly for isoproterenol vs. vehicle control, where $\bar{x}$ is the mean of normalized gene counts within a cell type for a particular gene. Effect size was calculated using Cohen’s d statistic,

$$\frac{{\bar{x}}_{night}-{\bar{x}}_{day}}{\sqrt{\frac{(n_{night}-1)s_{night}^{2}+(n_{day}-1)s_{day}^{2}}{n_{night}+n_{day}-2}s}}$$

where $\bar{x}$ is the mean of normalized gene counts within a cell type for a particular gene, *s* is the standard deviation for those counts, and *n* is the number of cells within that cell type. Effect size was similarly calculated for isoproterenol vs. vehicle control.

Data visualizations were prepared as follows: The dendrogram (Fig 1, S24 Fig) was generated using the BuildClusterTree function in Seurat, using ~1,500 highly variable genes returned by FindVariableGenes during clustering. Dotplots in Supporting Information (S1–S23 Figs) were generated using the DotPlot function in Seurat. The color intensity of each dot represents the average expression level of a given gene in a given cell type, converted to Z-scores. The size of the dot represents the fraction of cells within a cell type identity that express the given gene. Violin plots (Figs 1 and 3) were generated using the VlnPlot function in Seurat, or the ggplot2 package for R [61]. Marker heatmaps (Figs 3 and 4) were generated using the DoHeatMap function in Seurat. Differential expression heatmaps and Venn diagrams were generated using the ggplot2 and VennDiagram packages for R, respectively. Boxplots (S5 Fig) were generated using ggplot2.

### Immunohistochemical analysis

Fixed, embedded pineal glands were sectioned on a Leica CM3050S cryostat (Leica Biosystems; Nussloch, Germany) into 16 μm cryosections mounted on Superfrost Plus slides (Fisher Scientific; Pittsburgh, PA) and 30 μm sections floating in PBS. Sections were processed through an immunohistochemical protocol as described previously [62]. Briefly, sections were blocked and permeabilized for 1 hour in Carrier solution: PBS, pH 7.4, containing 0.3% Triton X-100, 1% goat or donkey serum, and 0.5% bovine serum albumen (EMD Millipore; Billerica, MA). Sections were incubated in primary antibodies diluted in Carrier solution (S4 Table) at 4°C for 18 hours, followed by extensive washing. Secondary antibodies (S4 Table) were applied for 1 hour at room temperature. All sections were incubated in 300 nM 4’,6-diamino-2-phenylindole (DAPI) during a final rinse in PBS. Floating sections were mounted onto gelatin-coated slides, air dried and preserved under #1.5 German cover glass using Prolong Diamond mountant (Invitrogen; Carlsbad,CA). Cryosections were air dried briefly before application of mountant and coverglass. Labeled sections were imaged on an inverted LSM 780 confocal microscope with a Plan-Apochromat 20X, 0.8NA lens (Zeiss; Thornwood, NY) in Z-stacked (15–20 μm) tiles or single planes. Single-plane images were acquired using near-saturating settings to optimize visualization of cells with low expression levels of the target antigen; tiles were stitched, and stacks were rendered as maximum intensity projections. Image intensities were optimized in Photoshop (Adobe; San Jose, CA). Antibodies were tested using brain sections in parallel trials to ensure known cell-type specificity and to provide positive technical controls. Negative controls (no primary antibody; no primary or secondary antibody) were included in each experiment to determine the level of non-specific secondary antibody binding and sample autofluorescence. In all cases presented, autofluorescence was minimal and non-specific signal above autofluorescence was absent. The presented results are representative of three or more repeated trials.

## Supporting information

S1 Dataset Differential expression analysis of rat pineal gland single cells between daytime and nighttime. (XLSX)

S2 Dataset Differential expression analysis of rat pineal gland single cells between isoproterenol treated rats and vehicle control. (XLSX)

S3 Dataset Differentially expressed genes between the daytime and nighttime rat pineal gland. (XLSX)

S4 Dataset Differentially expressed genes between isoproterenol and vehicle control treated rat pineal gland. (XLSX)

S1 Fig Relative expression of pinealocyte and astrocyte markers. (PDF)

S2 Fig Relative expression of catecholamine and cholinergic receptor transcripts. (PDF)

S3 Fig Relative expression of oxidative phosphorylation and RP transcripts. (PDF)

S4 Fig Relative expression of G-Protein subunit transcripts. (PDF)

S5 Fig Expression of specific genes included in Fig 3 between α- and β-pinealocytes. (PDF)

S6 Fig Immunohistochemical analysis of the pineal gland reveals cell type-specific patterns of expression. (PDF)

S7 Fig Regional differences in the density of S100b-positive and Gfap-postitive cells. (PDF)

S8 Fig Relative expression of microglia markers. (PDF)

S9 Fig Relative expression of VLMC and endothelial cell markers. (PDF)

S10 Fig Relative expression of cadherin and gap junction transcripts. (PDF)

S11 Fig Relative expression of glutamate and GABA receptor transcripts. (PDF)

S12 Fig Relative expression of purinergic receptor transcripts. (PDF)

S13 Fig Relative expression of calcium channel transcripts. (PDF)

S14 Fig Relative expression of potassium channel transcripts. (PDF)

S15 Fig Relative expression of adenylate cyclase and cyclic nucleotide phosphodiesterase transcripts. (PDF)

S16 Fig Relative expression of secretion related transcripts. (PDF)

S17 Fig Relative expression of ephrin ligand and receptor transcripts. (PDF)

S18 Fig Relative expression of Tfna ligand and receptor transcripts. (PDF)

S19 Fig Relative expression of transcription factor transcripts. (PDF)

S20 Fig Relative expression of aquaporin transcripts. (PDF)

S21 Fig Relative expression of glutamate transporter transcripts. (PDF)

S22 Fig Relative expression of clock gene transcripts. (PDF)

S23 Fig Relative expression of lipoxygenase and phospholipase transcripts. (PDF)

S24 Fig Transcriptomic characterization of nighttime pineal gland, batch effects, and QC. (PDF)

S1 Table Number of single cells profiled by cell type, day/night experiment. (PDF)

S2 Table Number of single cells profiled by cell type, isoproterenol experiment. (PDF)

S3 Table Annotation adjustment of 3’ ends of select genes. (PDF)

S4 Table Immunohistochemical reagents. (PDF)

S5 Table Differentially expressed genes overlapping between night/ isoproterenol treatment and day/vehicle control treatment. (PDF)
