# Supplementary material for: Single-cell RNA sequencing of the mammalian pineal gland identifies two pinealocyte subtypes and cell type-specific daily patterns of gene expression
Source: PLoS One. 2018 Oct 22;13(10):e0205883. doi: 10.1371/journal.pone.0205883 (PMC6197868; doi:10.1371/journal.pone.0205883)
Supplement: S5 Fig — (PDF) [file pone.0205883.s009.PDF]

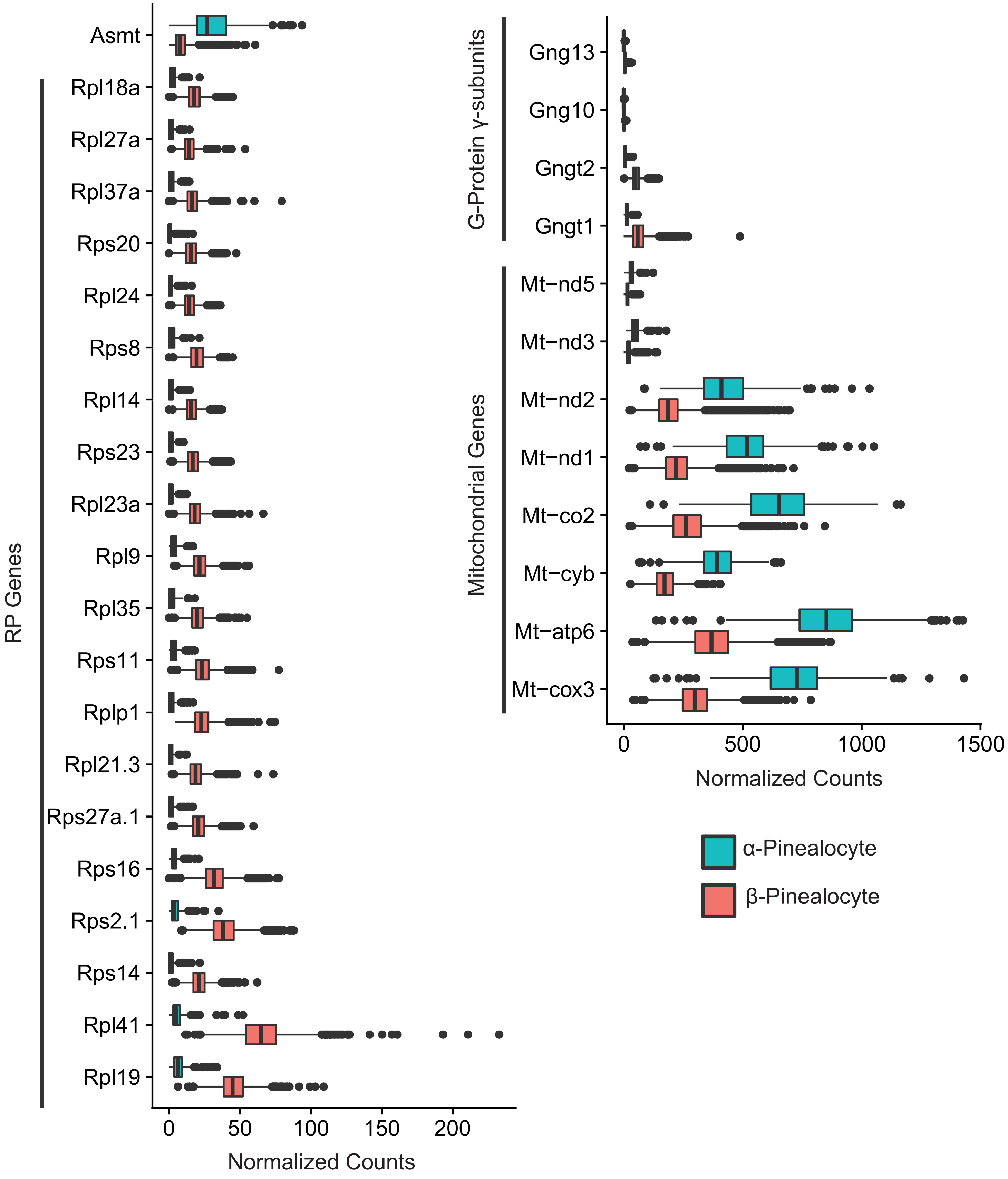

S5 Fig. Expression of specific genes included in Figure 3 between α- and β-pinealocytes. Boxplots include normalized counts from all cells in either cell type. Black dots are outliers. Data points are marked as outliers if they are 1.5X the inter quartile range above the third quartile or below the first quartile. All cells of each type are included (α-pinealocyte, n=275; β-pinealocyte, n=4,822).
