# Supplementary material for: Single-cell RNA sequencing of the mammalian pineal gland identifies two pinealocyte subtypes and cell type-specific daily patterns of gene expression
Source: PLoS One. 2018 Oct 22;13(10):e0205883. doi: 10.1371/journal.pone.0205883 (PMC6197868; doi:10.1371/journal.pone.0205883)
Supplement: S6 Fig — (PDF) [file pone.0205883.s010.pdf]

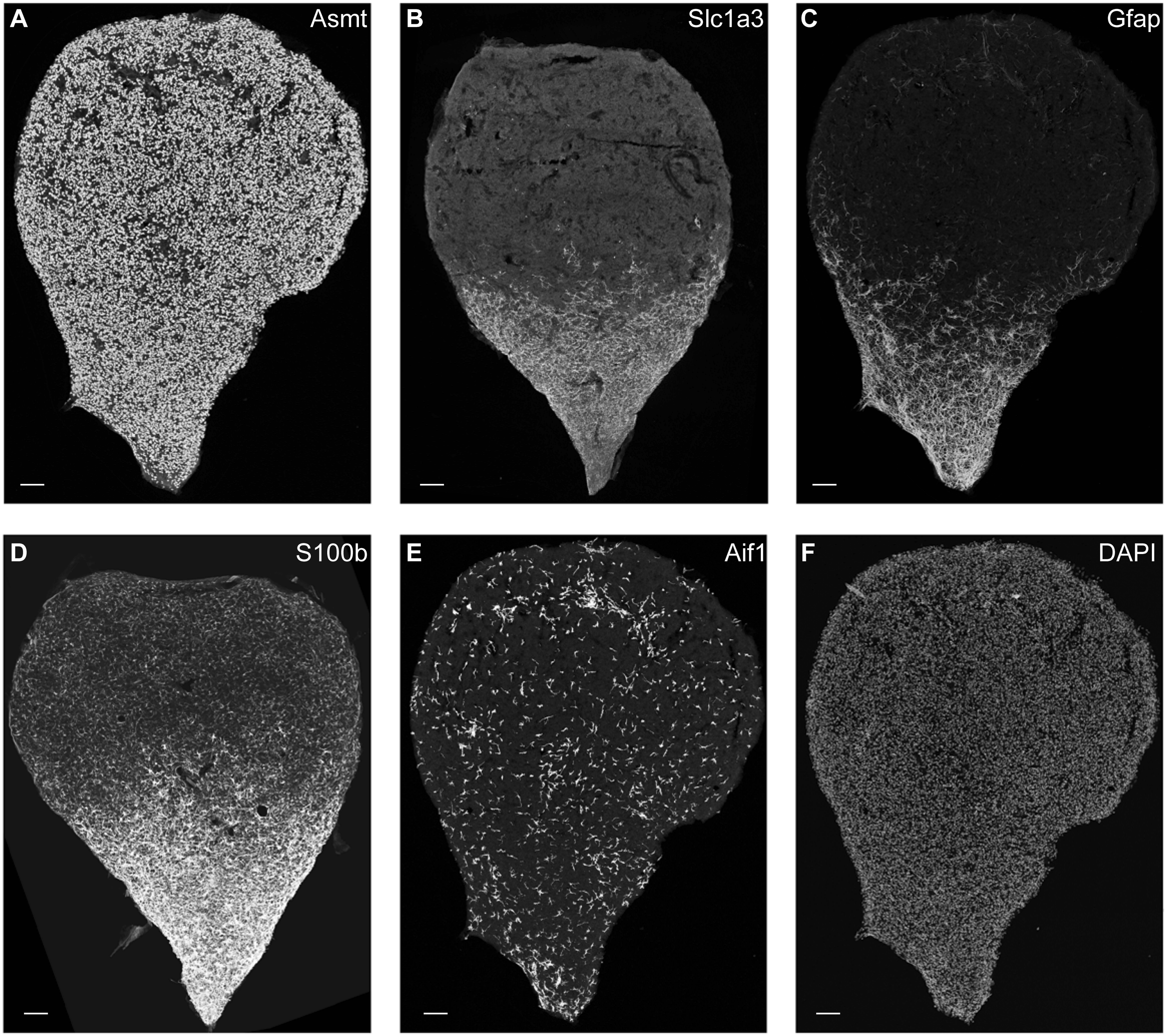


**S6 Fig. Immunohistochemical analysis of the pineal gland reveals cell type-specific patterns of expression.** Maximum intensity projections taken from immunohistochemical sections through the rat pineal gland midline with the rostral stalk origin at the bottom. Scale bar = 100 μm.
**(A)** Asmt-positive pinealocytes are uniformly distributed. **(B)** Slc1a3-positive γ-astrocytes are most abundant in rostral region near the stalk. **(C)** Gfap-positive γ-astrocytes are most abundant in rostral region near the stalk. **(D)** S100b-positive astrocytes are most abundant in the rostral region and appear elsewhere with distinctly lower density and expression strength. **(E)** Aif1-positive microglia are unevenly distributed throughout pineal gland at low density. **(F)** DAPI stain indicates a uniform distribution of cells throughout the gland.
