# Supplementary material for: Single-cell RNA sequencing of the mammalian pineal gland identifies two pinealocyte subtypes and cell type-specific daily patterns of gene expression
Source: PLoS One. 2018 Oct 22;13(10):e0205883. doi: 10.1371/journal.pone.0205883 (PMC6197868; doi:10.1371/journal.pone.0205883)
Supplement: S7 Fig — (PDF) [file pone.0205883.s011.pdf]

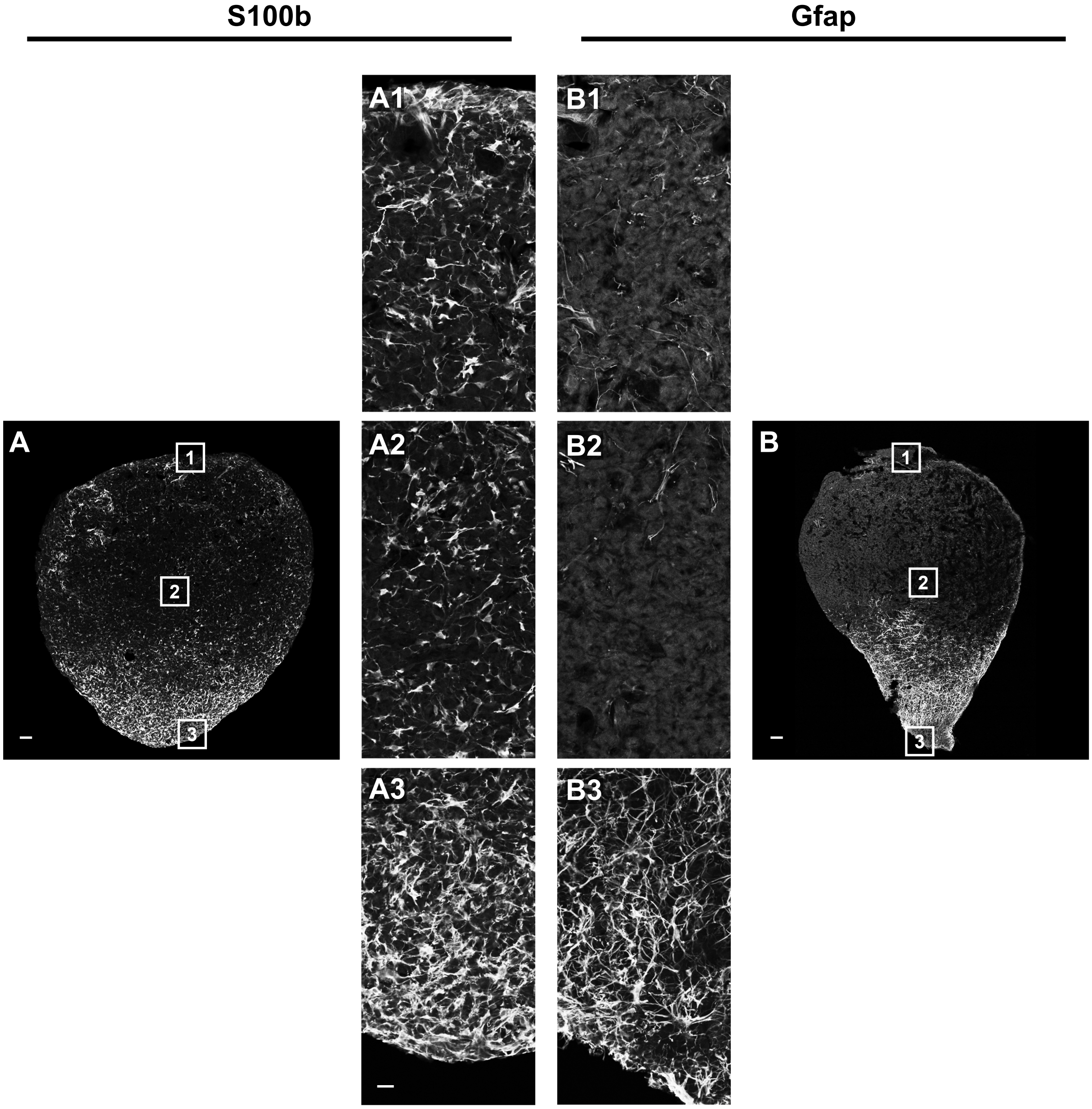

S7 Fig. Regional differences in the density of S100b-positive and Gfap-postitive cells. Images were taken from immunohistochemical sections through the rat pineal gland midline with the rostral stalk origin at the bottom. **(A-B)** Full gland images acquired as tiled stacks, rendered as maximum intensity projections. Scale bar = 100 *μ*m. **(A1-3, B1-3)** Single-plane images aquired from regions designated in A and B. Scale bar = 20 *μ*m. **(A, A1-3)** S100b-positive astrocytes are most abundant in the rostral region of the gland and appear elsewhere with distinctly lower density and expression strength. **(B, B1-3)** Gfap-positive γ-astrocytes are most abundant in the rostral region near the stalk.
