# Supplementary material for: Single-cell RNA sequencing of the mammalian pineal gland identifies two pinealocyte subtypes and cell type-specific daily patterns of gene expression
Source: PLoS One. 2018 Oct 22;13(10):e0205883. doi: 10.1371/journal.pone.0205883 (PMC6197868; doi:10.1371/journal.pone.0205883)

S11 Fig. Relative expression of glutamate and GABA receptor transcripts. Color intensity represents the z-scored average of normalized counts for a given gene across all cells. Dot size represents the fraction of cells within a cell type that express a given gene (see legend at bottom). All day and night samples included (N=13,607). (+) symbol above dot indicates transcript upregulation at night, (-) indicates upregulation during the day (p<0.01, Wilcoxon rank sum; effect size ≥ 0.35, fold change ≥ 2.0, expressed in ≥ 15.0% of cells).

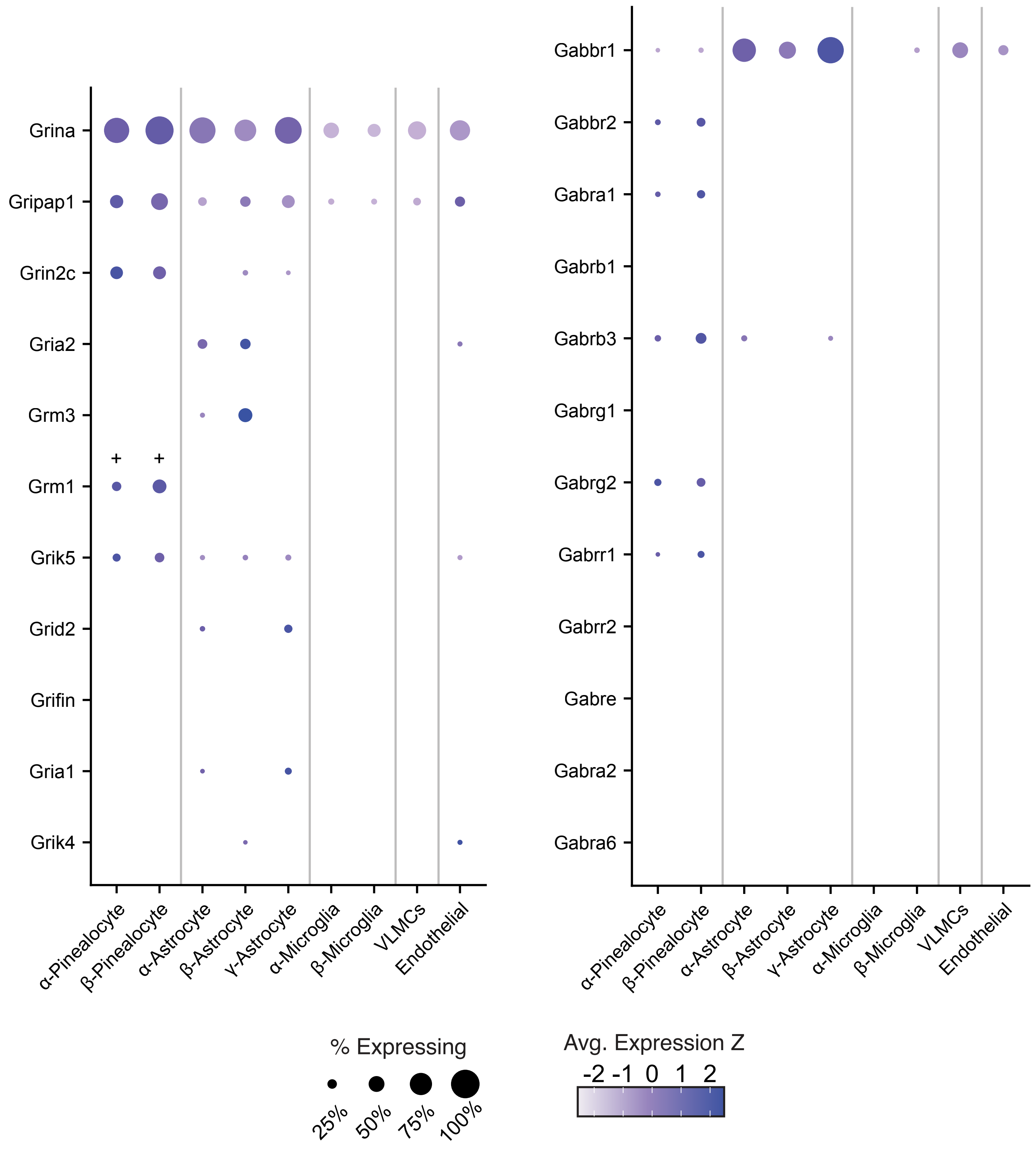

Supplement: S11 Fig — (PDF) [file pone.0205883.s015.pdf]
