# Supplementary material for: Single-cell RNA sequencing of the mammalian pineal gland identifies two pinealocyte subtypes and cell type-specific daily patterns of gene expression
Source: PLoS One. 2018 Oct 22;13(10):e0205883. doi: 10.1371/journal.pone.0205883 (PMC6197868; doi:10.1371/journal.pone.0205883)
Supplement: S24 Fig — (PDF) [file pone.0205883.s028.pdf]

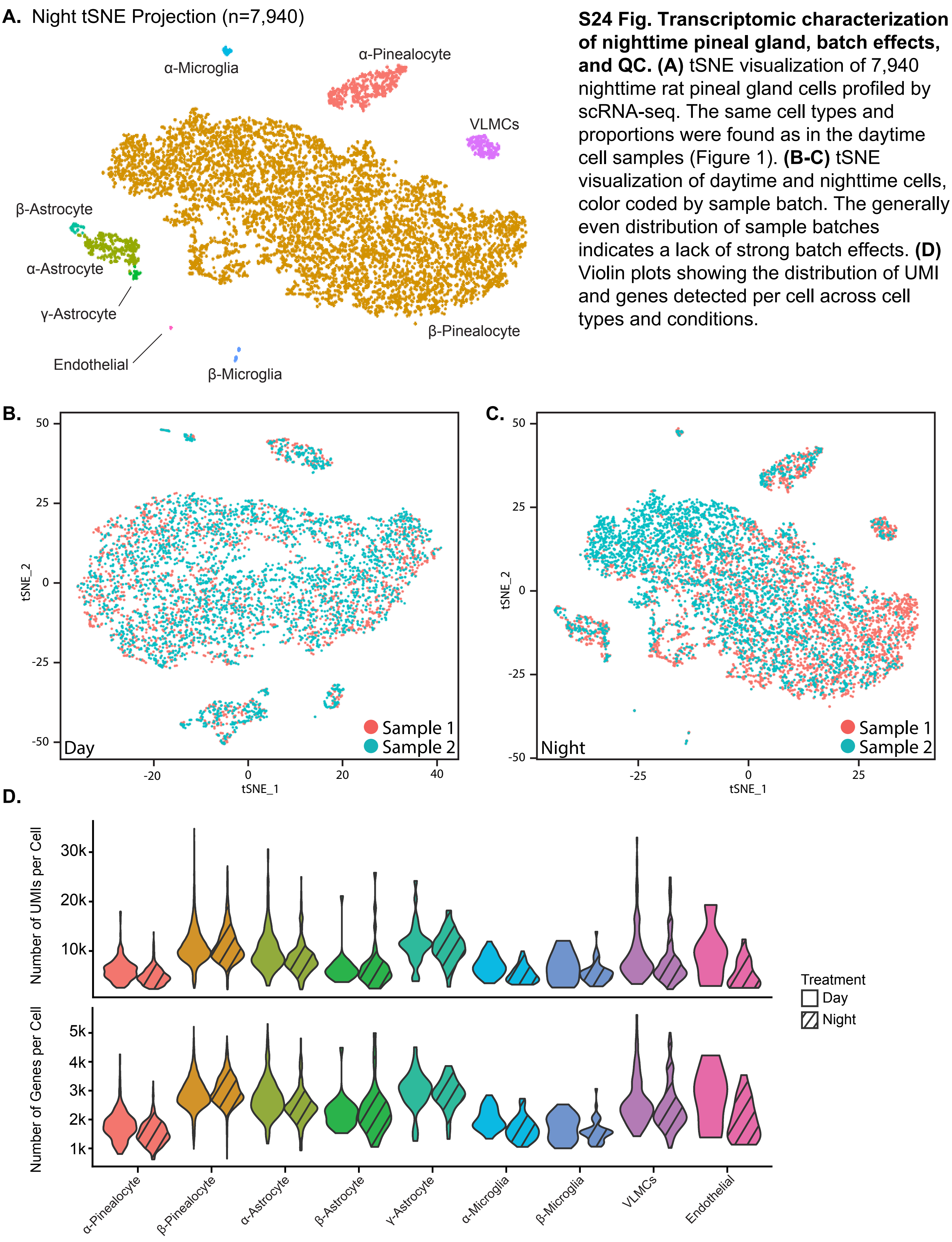

S24 Fig. Transcriptomic characterization of nighttime pineal gland, batch effects, and QC. (A) tSNE visualization of 7,940 nighttime rat pineal gland cells profiled by scRNA-seq. The same cell types and proportions were found as in the daytime cell samples (Figure 1). **(B-C)** tSNE visualization of daytime and nighttime cells, color coded by sample batch. The generally even distribution of sample batches indicates a lack of strong batch effects. **(D)** Violin plots showing the distribution of UMI and genes detected per cell across cell types and conditions.
