# Supplementary material for: Single-cell RNA sequencing of the mammalian pineal gland identifies two pinealocyte subtypes and cell type-specific daily patterns of gene expression
Source: PLoS One. 2018 Oct 22;13(10):e0205883. doi: 10.1371/journal.pone.0205883 (PMC6197868; doi:10.1371/journal.pone.0205883)
Supplement: S1 Table — (PDF) [file pone.0205883.s029.pdf]

S1 Table. Number of single cells profiled by cell type, day/night experiment

**Cell Type**	**Day**	**Night**
α-Pinealocyte	257	422
β-Pinealocyte	4822	6848
α-Astrocyte	339	301
β-Astrocyte	29	43
γ-Astrocyte	32	46
α-Microglia	44	47
β-Microglia	25	46
VLMCs	113	168
Endothelial	6	19
**Total**	5667	7940
