# Supplementary material for: Single-cell RNA sequencing of the mammalian pineal gland identifies two pinealocyte subtypes and cell type-specific daily patterns of gene expression
Source: PLoS One. 2018 Oct 22;13(10):e0205883. doi: 10.1371/journal.pone.0205883 (PMC6197868; doi:10.1371/journal.pone.0205883)
Supplement: S2 Table — (PDF) [file pone.0205883.s030.pdf]

S2 Table. Number of single cells profiled by cell type, isoproterenol experiment

Cell Type	Vehicle	Isoproterenol
α-Pinealocyte	121	98
β-Pinealocyte	1320	1726
Astrocyte	102	118
Microglia	7	10
VLMCs	21	30
Endothelial	5	14
**Total**	1576	1996
