# Supplementary material for: Single-cell RNA sequencing of the mammalian pineal gland identifies two pinealocyte subtypes and cell type-specific daily patterns of gene expression
Source: PLoS One. 2018 Oct 22;13(10):e0205883. doi: 10.1371/journal.pone.0205883 (PMC6197868; doi:10.1371/journal.pone.0205883)
Supplement: S3 Table — (PDF) [file pone.0205883.s031.pdf]

S3 Table. Annotation adjustment of 3’ ends of select genes. The original and revised 3’ exon boundaries for adjusted genes are given. See methods for details.

Gene	chromo-some	Original 3'UTR end in rn6	Revised 3'UTR end in rn6	Strand	Extension (bp)
**Adrb1**	1	277,538,988	277,540,348	+	1,360
**Chrnb2**	2	189,088,566	189,085,442	-	3,124
**Clcn4**	X	25,080,416	25,082,550	+	2,134
**Clic4**	5	153,568,933	153,565,828	-	3,105
**Crx**	1	77,745,289	77,743,937	-	1,352
**Gabrb3**	1	113,265,374	113,268,943	+	3,569
**Gabrg2**	10	27,093,030	27,090,901	-	2,129
**Hcn1**	2	50,499,810	50,502,563	+	2,753
**Hcn2**	7	12,851,729	12,851,162	-	567
**Kcnab2**	5	169,572,704	169,570,319	-	2,385
**Kcnq4**	5	139,627,575	139,625,789	-	1,786
**Map2k4**	10	52,196,116	52,193,754	-	2,362
**Map2k7**	12	2,548,224	2,546,146	-	2,078
**Map4k5**	6	92,136,975	92,136,227	-	748
**Mapk1**	11	88,211,647	88,208,129	-	3,518
**P2ry4**	X	70,426,946	70,421,608	-	5,338
**Ppm1a**	6	94,484,524	94,484,976	+	452
**Ppp1r7**	9	100,504,082	100,511,241	+	7,159
**Prkaa2**	5	124,574,072	124,568,840	-	5,232
**Prkacb**	2	252,605,306	252,602,309	-	2,997
**Prkar2a**	8	117,545,567	117,548,763	+	3,196
**Prkce**	6	9,973,401	9,976,191	+	2,790
**Prkd1**	6	71,035,788	71,035,025	-	763
**Rbp3**	16	10,286,241	10,291,917	+	5,676
**Scn2b**	8	49,427,557	49,431,756	+	4,199
**Scn7a**	3	52,775,761	52,764,946	-	10,815
**Scn8a**	7	142,683,667	142,688,451	+	4,784
**Tab2**	1	1,999,573	1,999,101	-	472
