# Supplementary material for: Single-cell RNA sequencing of the mammalian pineal gland identifies two pinealocyte subtypes and cell type-specific daily patterns of gene expression
Source: PLoS One. 2018 Oct 22;13(10):e0205883. doi: 10.1371/journal.pone.0205883 (PMC6197868; doi:10.1371/journal.pone.0205883)
Supplement: S4 Table — (PDF) [file pone.0205883.s032.pdf]

S4 Table. Immunohistochemical reagents

Target Protein	Antigen	Description	Source	Dilution	Secondary*
S100b	Purified S100 from cow brain	Rabbit polyclonal	DAKO Z0311	1:1000	AlexaFluor 488 Goat anti-rabbit IgG
Slc1a3 (Eaat1/Glast)	Synthetic 20 AA C-terminus rat EAAT1 peptide	Rabbit polyclonal	Abcam ab416	1:500	AlexFluor 555 Goat anti-Rabbit IgG
Glial fibrillary acidic protein (Gfap)	Recombinant full-length protein corresponding to human GFAP	Chicken polyclonal	Abcam ab4674	1:1000	DyLight 488 Donkey anti-Chicken IgY
Acetylserotonin-O-methyltransferase (Asmt)	Synthetic peptide EGWERQASDYRNLA	Rabbit polyclonal	Dr. David Klein	1:1000	633 AlexaFluor Donkey anti-Rabbit IgG
Aif1 (Iba1)	Synthetic peptide corresponding to human Iba1 (aa135-147) C-TGPPAKKAISELP	Goat polyclonal	Abcam ab5076	1:500	555 AlexaFluor Donkey anti-Goat IgG

*Source: Invitrogen, diluted 1:1000
