# Supplementary material for: Single-cell RNA sequencing of the mammalian pineal gland identifies two pinealocyte subtypes and cell type-specific daily patterns of gene expression
Source: PLoS One. 2018 Oct 22;13(10):e0205883. doi: 10.1371/journal.pone.0205883 (PMC6197868; doi:10.1371/journal.pone.0205883)
Supplement: S5 Table — (PDF) [file pone.0205883.s033.pdf]

S5Table. Differentially expressed genes overlapping between night/isoproterenol treatment and day/vehicle control treatment.

	**Cell Types**		
**Treatment**	α-Pinealocytes	β-Pinealocytes	Astrocytes
Night and Isoproterenol	Aanat, Abca1, Asl, Atp7b, B3gnt8, Camk1g, Dclk3, Dnm2, Drd4, Etnk1, Farp2, Fcer1a, Frmpd1, Fry, Galnt16, Gdf15, Hspa1b, Hspa5, Irak2, Itpr1, Kcnab2, Kctd3, Lnx1, Mat2a, Mcam, Mmd2, Nptx1, Pomgnt1, Pqlc1, Ptch1, Reep2, Rpap1, Sik2, Slc15a1, Srrm4	Aanat, Abca1, Abcf1, Asl, Atp7b, B3gnt8, Bud31, Cacna1g, Cacna1h, Calhm2, Camk1g, Cd24, Cd8a, Cdc5l, Cerk, Cmip, Cnot11, Coq10a, Coq10b, Creb3, Crem, Dclk1, Dclk3, Ddit4, Decr1, Dgat2, Dnajb1, Dnm2, Drd4, Eif1b, Emd, Endog, Eprs, Etnk1, Exosc5, Fam161a, Fbxo6, Fcer1a, Fdx1, Fkbp5, Fndc4, Frmpd1, Fry, G0s2, Galnt16, Gdf15, Gjc2, Gmnn, Grm1, Gstt3, Gtpbp4, Gxylt1, Herpud1, Hyal2, Id1, Ifngr1, Impdh2, Irak2, Irs2, Kcnq4, Kctd3, Lamb1, Lamb3, Lcp1, Lmo1, Lnx1, Lrrc73, Lxn, Man2a1, Mapk10, Marcks, Marcksl1, Mbnl2, Mcam, Mettl8, Mmd2, Mt1, Ncald, Nphp4, Nptx1, Nradd, Nudt19, Oasl, Osbp2, Padi4, Parvb, Pcna, Pde10a, Pde4b, Pmepa1, Pmvk, Pomgnt1, Pqlc1, Prdx1, Ptch1, Ptp4a3, Qsox1, Rab3a, Rabepk, Rcan1, Reep2, RGD1310852, RGD1562747, Ribc2, Robo2, Sash1, Sik2, Slbp, Slc15a1, Slc3a2, Slc4a4, Slc7a5, Srrm4, Srsf3, Tbc1d1, Tc2n, Thap4, Them6, Thrb, Tmem117, Top1, Tpcn1, Tspyl4, Ttc8, Upf3b, Usp16, Vps4b, Wfikkn2, Wipi2, Wnt10a	Aanat*, Ednrb, Slc15a1*
Day and Vehicle Control	Acsl1, Ankrd27, Arf5, Ccl9, Cdc25a, Cdh2, Cfl2, Depdc7, Dhrs3, Eef1a2, Emc7, Extl2, Fkbp2, Frmd4b, Gemin8, Gng13, Gucy1a3, Hook1, Hs3st2, Icmt, Idh1, Kdm4c, Klhdc8b, Kpna2, LOC100361854, Mrps27, Nim1k, Pax4, Pdc, Pid1, Ptpmt1, Ptpn21, Rdh11, Reep6, Rgs20, Rrp1b, Sgk1, Snrpd3, Stxbp1, Tcta, Tmed1, Ush2a, Zfp444	Ankrd27, Anks1a, Anks6, B4galt5, Casp7, Ccl9, Cdc25a, Cdh2, Cfl2, Cpt1a, Depdc7, Dgkd, Dyrk1b, Dyrk2, Eef1a1, Eps8, Extl2, Frmd4b, Glce, Gucy1a3, Hdac5, Hook1, Hs3st2, Kdm3b, Kdm4c, Klhdc8b, Lpcat1, Mrps27, Nim1k, P2ry4, Pid1, Pla2r1, Podxl, Ptpn21, Rgs20, Rnf19b, Rrp1b, Rtn4rl1, Sgk1, Slc39a8, Stard7, Tcta, Tle1, Tmed1, Tmem65, Ush2a, Vac14, Wdr70, Zc3h14, Zfp143	

(*) Detection of differential expression of *Aanat* and *Slc15a1* in astrocytes is thought to arise from ambient mRNA contamination from lysed pinealocytes, as discussed in the main text.
